# Pharmacological Studies on Traditional Plant-Based Remedies

**DOI:** 10.3390/biomedicines9030315

**Published:** 2021-03-19

**Authors:** Giuseppe Lucariello, Donatella Cicia, Raffaele Capasso

**Affiliations:** 1 Department of Pharmacy, School of Medicine and Surgery, University of Naples Federico II, 80131 Naples, Italy; giuseppe.lucariello@unina.it (G.L.); donatella.cicia@unina.it (D.C.); 2 Department of Agricultural Sciences, University of Naples Federico II, 80055 Portici, Italy

For years, plant-based remedies have been used as a traditional practice to treat and prevent a broad range of diseases. During the past decade, natural therapies have regained public attention, and, to date, great interest has caught on, as demonstrated by the elevated number of new studies concerning this topic, and by the high funds earmarked every year on medicinal plants. Several reasons contribute to this renovated attention on herbal remedies, among which we can include: the prospect to study a high quantity of unexplored botanicals species; an eco-friendly and cost-efficient approach in terms of research, isolation, and production; the possibility to discover new antimicrobial natural products, that can face the current spreading of antibiotic resistance; the demanding need to reveal potential side effects and interactions of the most widely used natural products with concomitant drug therapies.

In this editorial, we have compiled 20 articles about this study area, summarized as below.

Cordeiro et al. studied the antinociceptive and anti-inflammatory effects of *Stevia serrata* Cav. (Asteraceae) essential oil (EO) and the mechanism of action using opioid and cholinergic antagonists (naloxone and atropine, respectively) and the nitric oxide synthase inhibitor (N-omega-nitro-L-arginine methyl ester, L-NAME).Their work suggests that essential oil of *S. serrata* presents an antinociceptive effect mediated, at least in part, through activation of opioid, cholinergic and nitrergic pathways [1]. *Piper sylvaticum* Roxb, is traditionally used by the indigenous people of tropical and subtropical countries like Bangladesh, India, and China for a variety of chronic diseases. Adnan et al. in their study tested the metabolites extracted (methanol) from the leaves and stems of *P*. *sylvaticum*, showing a reduction of anxiety-like behavior in vivo and a moderate antioxidant activity in vitro [2]. The Malaysian herb *Orthosiphon stamineus* is a traditional remedy that possesses anti-inflammatory, anti-oxidant, and free-radical scavenging abilities, all of which are known to protect against Alzheimer’s disease (AD). With their research, Retinasamy et al. demonstrated an improved effect of *O. stamineus* ethanolic extract on memory in rat, and hence, could serve as a potential therapeutic target for the treatment of neurodegenerative diseases such as AD [3]. Terpenoids are natural plant-derived products that are used to treat a broad range of human diseases, including airway infections and inflammation. However, pharmaceutical applications of terpenoids against bacterial infection remain challenging due to their poor water solubility. Kaltschmidt et al. perfectioned the preparation of terpenoid-invasomes with selective activity against *S. Aureus*. They also performed characterization by cryo-transmission electron microscopyand demonstrated that, particularly thymol-invasomes, show a strong selective activity against Gram-positive bacteria [4]. Salomè et al. evaluated the antinociceptive and anti-inflammatory activities of the essential oil (EO) of *Aristolochia trilobata* and its main ingredient the sulcatyl acetate (SA), they studied the mechanism of antinociceptive activity being evaluated in presence of opioid, cholinergic receptor antagonists (naloxone and atropine), or nitric oxide synthase inhibitor (L-NAME). EO and SA present peripheral and central antinociceptive and anti-inflammatory effects, mediated by inhibition of inducible nitric oxide synthase (iNOS) and spleen tyrosine kinases (Syk) expression [5]. Chemically nickel oxide nanoparticles (NiONP) involve the synthesis of some toxic products for different microbial agents and microalgae by producing reactive oxygen species (ROS), inducing oxidative stress and releasing (Ni^2+^) inside the cell, which restrict their biological applications. Iqbal et al. developed a chemistry method for the fabrication of NiONPs using fresh leaf broth of *Rhamnus triquetra* (RT), making them an attractive and eco-friendly alternative, that also showed potential in vitro biological activities [6].In his review, Habtemariam scrutinizedin vitro, in vivo, and clinical outcomes of *Trametes versicolor (L.)* polysaccharides which are thought to being useful as adjuvant therapy for cancer [7].Park study related about anti-anaphylactic activity of isoquercitin (Quercetin-3-O-β-d-Glucose) (IQ) in cardiovascular systems of experimental animals, like rats and pigs. Overall, this study provided evidence for the beneficial effect of IQ on cardiac anaphylaxis, thus suggesting its potential applications in the treatment and prevention of related diseases [8]. Picciolo et al. evaluated the therapeutic potential of *β*-Caryophyllene (BCP), a cannabinoid receptor 2 (CB2) agonist, in an in vitro model of oral mucositis, exploring the human gingival fibroblasts (GF), and human oral mucosa epithelial cells (EC) with an inflammatory phenotype representing a valuable experimental paradigm. BCP blunted the lipopolysaccharides (LPS)-induced inflammatory phenotype and this effect was reverted by the CB2 antagonist AM630. These results suggest that CB2 receptors are an interesting target to develop innovative strategies for oral mucositis [9]. Ikarashi et al., starting from previously data that suggest an inhibitory effect byergosterol on bladder carcinogenesis, elucidated its molecular mechanism using a rat model of N-butyl-N-(4-hydroxybutyl)-nitrosamine-induced bladder cancer. They also analyzed various aspects of the cell cycle, inflammation-related signaling, and androgen signaling, suggesting that ergosterol inhibits bladder carcinogenesis [10]. Georgieva et al. demonstrated that hemocyanins isolated from *H. aspersa*, *H. lucorum,* and *R. venosa*, as well as the mucus from *H. aspersa* exert an antitumor activity in vitro against colorectal carcinoma cell line HT-29, reducing cell viability with a mechanism that includes the induction of apoptosis [11]. Ashrafizadeh et al. reviewed the therapeutic effects of resveratrol, shading light on its possible impact on the tumor growth factor beta (TGF-beta) signaling pathway. Interestingly, resveratrol inhibits both upstream (such as microRNAs (miR)) and downstream mediators of TGF-beta signaling (small mother against decapentaplegic (SMAD), programmed cell death protein 1 (known asPD-1) andepithelial mesenchymal transition (EMT)). Via the down regulation of this pathway, resveratrol exerts its anti-fibrotic, anti-tumor, neuroprotective, lung protective, and anti-diabetics effects [12]. Álvarez-Martínez et al. reviewed the activity of the most representative antimicrobial products of natural origin. Mostnatural products (NP), do not have sufficient therapeutic power to be used in monotherapy against antibiotic resistant bacteria, but some of them have shown synergistic capacity with traditional antibiotics [13]. Casili et al. demonstrated anti-inflammatory properties of luteolin in a model of periodontitis induced by LPS in rats. Based on these results, luteolin implementation could represent a support to the traditional pharmacological approach for periodontitis [14]. Rahman et al. analyzed the role of natural compounds in the modulation of autophagy pathway in cancer prevention and treatment, neurodegenerative and cardiovascular diseases. Mammalian target of rapamycin (mTOR) and adenosine monophosphate-activated protein kinase (AMPK) are the leading regulatory path way of autophagy and they are known targets for natural compounds such as resveratrol, curcumin, antroquinonol and many others [15]. Sbrini et al. investigated the effect of the chronic oral treatment for 10 days with a phytosomal preparation containing *Centellaasiatica* and *Curcumalonga* on brain-derived neurotrophic factor (BDNF) levels in prefrontal cortex of adult rats. The phytosome ameliorates brain plasticity, enhancing mTOR-S6 regulated transcription of proteins involved in memory processes, suggesting that this preparation can be used as a supporting therapy in subjects with memory and cognitive disfunction [16]. Behl et al. discussed withaferin A (WA) pharmacokinetics, synergistic combination, and biological activities. This review highlighted that WA is a promising anticancer compound, but its benefits include also AD, cardioprotective, neuroprotective, osteoporotic, and antiviral effects. Moreover, according to pharmacokinetics studies, it can be used to design drug delivery systems [17]. Kim et al. investigated whether kahweol exerts a protective effect against cisplatin-induced renal injury. The results show that kahweol inhibits immune cell accumulation presumably through down regulation of vascular adhesion molecules, suggesting that it can be a potential preventive agent against cisplatin-induced acute kidney injury, enabling the use of a high dose of cisplatin [18]. Rapa et al. evaluated the effect of plumericin to improve intestinal epithelial barrier function both in intestinal epithelial cells in vitro, and in vivo in a model of dinitrobenzene sulfonic acid (DNBS) induced colitis. This study provided evidence that plumericin improves the expression of junctions’ proteins in the epithelial cells, reducing also apoptotic parameters, and enhancing actin cytoskeleton rearrangement. In vivo experiments sustain this evidence, thus supporting the pharmacological potential of plumericin as an adjuvant in inflammatory bowel diseases (IBD) [19].In their review, Devi et al. provided an insight into the potential role of flavonoids against cellular stress response in neurodegenerative disorders. Flavonoids have the potential to reduce these exaggerated cellular stress responses in-turn preventing cell death. Further studies are needed to determine their clinical acceptance [20].

## Data Availability

Not applicable.
